# Therapeutic Interventions in Organophosphate Poisoning: An Umbrella Review of Systematic Reviews

**DOI:** 10.5811/westjem.50823

**Published:** 2026-05-19

**Authors:** Vivek Chauhan, Divyam Goyal, Suman Thakur, Sagar Galwankar, Tamas R. Peredy

**Affiliations:** * Indira Gandhi Medical College Shimla, Department of Medicine, Himachal Pradesh, India; † Maharishi Markandeshwar Institute of Medical Sciences and Research, Department of Medicine, Haryana, India; ‡ Indira Gandhi Medical College Shimla, Department of Microbiology, Himachal Pradesh, India; § Florida State University College of Medicine Emergency Medicine, Sarasota Memorial Hospital, Department of Emergency Medicine, Sarasota, Florida

## Abstract

**Introduction:**

Organophosphate (OP) poisoning is a significant global health issue, particularly in tropical regions. Despite established treatments such as atropine and oximes, the effectiveness of other interventions remains uncertain. This umbrella review is a critical synthesis of evidence from systematic reviews and meta-analyses on OP self-poisoning.

**Methods:**

Following the Preferred Reporting Items for Systematic reviews and Meta-Analyses guidelines, we conducted a review of systematic reviews and meta-analyses published up to January 2025. Databases searched included PubMed, Epistemonikos, and the Cochrane Library. We performed quality assessment using A Measurement Tool to Assess Systematic Reviews, version 2 (AMSTAR-2), and applied the Grading of Recommendations Assessment, Development and Evaluation (GRADE) approach to evaluate evidence certainty.

**Results:**

Of 416 potential papers identified we assessed 27 for eligibility, of which 19 were included in the review. The papers evaluated 11 different interventions as an adjuvant to atropine. Oximes, although commonly used, showed neither benefit nor harm. The systematic reviews and meta-analyses on gastric lavage, plasma exchange with hemoperfusion, lipid emulsions, magnesium sulfate, penehyclidine, rhubarb, and xuebijing have reported significant reductions in mortality, but the evidence comes from very low-quality studies. Alkalinization was not found to be effective for OP poisoning. Evidence was limited by small sample size, inconsistent protocols, and geographical bias, with many studies originating from China.

**Conclusion:**

After careful scrutiny of evidence pooled by various systematic reviews and meta-analyses, we found that atropine remains the mainstay of treatment for OP self-poisoning. It may be supplemented with oximes, as recommended by the World Health Organization. Gastric lavage has doubtful efficacy and may even be harmful. Additionally, we recommend against the routine use of penehyclidine, rhubarb, xuebijing, hemofiltration, plasma exchange with hemoperfusion, lipid emulsions, magnesium sulfate, and alkalinization in the management of OP self-poisoning.

## INTRODUCTION

Organophosphate (OP) poisoning is a critical global health issue, particularly in agricultural regions, and it is associated with high morbidity and mortality rates.^1^ There is no universally accepted “international guideline” due to ongoing clinical controversies and variations in regional practices. Despite the absence of guidelines, the core principles of treatment are widely agreed upon. The first step involves decontamination, which focuses on preventing further exposure to both patients and healthcare workers. All contaminated clothing and bodily fluids should be carefully removed and disposed of safely, while patients should be thoroughly washed with soap and water.^2^ In cases where ingestion has occurred, gastric lavage may be considered if the patient presents within 1–2 hours and the airway is protected. Activated charcoal (50 grams [g], without cathartic) may be given orally or via a nasogastric tube to cooperative or intubated patients, particularly when presentation is early or toxicity is severe.^2^

Following decontamination, immediate stabilization and resuscitation based on the airway, breathing, and circulation is essential. This includes securing the airway, providing oxygen, and initiating ventilatory support when required. Once the patient is stabilized, antidote therapy forms the cornerstone of management. Atropine is administered to counteract muscarinic symptoms; in adults, the initial intravenous (IV) dose is 1–3 mg, while children receive 0.02 mg per kilogram (kg).^2^ Doses are titrated to achieve a clear chest on auscultation, resolution of bronchorrhea, and a heart rate > 80 beats per minute. If these targets are not reached within 3–5 minutes, the dose is doubled and repeated at the same interval until atropinization is achieved. In severe cases, very large cumulative doses, sometimes reaching hundreds of milligrams, may be required. Maintenance therapy is then provided through a continuous infusion at a rate of 10–20% of the total loading dose per hour.^2^

Oxime therapy is recommended along with atropine to reactivate acetylcholinesterase. Pralidoxime chloride is administered as an IV loading dose of 30 mg/kg over 20 minutes, followed by a continuous infusion of 8 mg/kg/hour; in adults this is commonly given as a 2 g loading dose followed by 500 mg/hour. Alternatively, obidoxime may be used, with a loading dose of 4 mg/kg (about 250 mg in adults) given over 20 minutes, followed by 750 mg every 24 hours or a continuous infusion at 0.5 mg/kg/hour. Oxime infusions are continued until the patient shows clinical recovery.^2^

Benzodiazepines are used when agitation or seizures occur, with IV administration preferred. Common regimens include diazepam 5–10 mg (0.05–0.3 mg/kg/dose), lorazepam 2–4 mg (0.05–0.1 mg/kg/dose), or midazolam 5–10 mg (0.15–0.2 mg/kg/dose).^2^ In addition, adjunctive measures such as magnesium sulfate have been studied and may improve outcomes, although their role remains supportive. Together, these principles—decontamination, stabilization, antidote therapy, and adjunctive care—form the accepted standard of management for acute OP poisoning across most clinical settings.

The mainstay of therapy for OP poisoning has been atropine and oximes, but in the past decade several systematic reviews have shown that oximes are ineffective, if not harmful.^3^,^4^ The treatment protocol for OP poisoning was reviewed in 2006 by Eddleston et al and published in the *British Medical Journal Clinical Evidence*.^5^ They categorized the evidence as 1) likely to be beneficial (atropine, benzodiazepines for OP-induced seizures, glycopyrrolate, and washing the poisoned person or removing contaminated clothing); 2) unknown effectiveness (activated charcoal, α-2 adrenergic receptor agonists, butyrycholinesterase replacement therapy, extracorporeal clearance, gastric lavage, and magnesium sulfate, milk or other home remedies immediately after ingestion, N-methyl-D-aspartate receptor antagonists, organophosphate hydrolases, oximes and sodium bicarbonate); 3) unlikely to be beneficial (cathartics); 4) likely to be ineffective or harmful (ipecac).^5^ This review included evidence from published systematic reviews and meta-analysis, randomized control trials (or cohort studies) until 2006. The authors used Grading of Recommendations Assessment, Development and Evaluation (GRADE) evaluation of evidence to give the level of evidence for each of the above interventions.^5^

**Population Health Research Capsule**

What do we already know about this issue?

*Atropine is the mainstay of treatment of organophosphate (OP) poisoning. Oximes have been found neither helpful nor harmful in the latest metanalysis. There are no other proven therapies for OP poisoning*.

What was the research question?

*To critically review and summarize the available evidence from published systematic reviews and metanalyses for the effect of various treatments on the outcomes of OP self-poisoning*.

What was the major finding of the study?

*Atropine remains the mainstay of treatment for OP self-poisoning. It may be supplemented with oximes as recommended by the World Health Organization*.

How does this improve population health?

*We recommend against the routine use of penehyclidine, rhubarb, xuebijing, hemofiltration, plasma exchange with hemoperfusion, lipid emulsions, magnesium sulfate and alkalinization in the management of OP self-poisoning*.

In recent years, evidence from the published systematic reviews and meta-analyses have shown the effectiveness of adjuvant therapies such as magnesium sulfate, penehyclidine, xuebijing, crude rhubarb, lipid emulsion, hemofiltration with hemoperfusion and plasma exchange plus hemoperfusion about which most physicians are unaware.^6^–^13^ There were no guidelines on OP poisoning management that reviewed the emerging evidence generated by the systematic reviews and meta-analyses conducted after 2006. Therefore, we conducted this umbrella review using GRADE and A Measurement Tool to Assess Systematic Reviews (AMSTAR) to analyze systematic reviews and meta-analyses and assess the quality of evidence generated by each of the published systematic reviews and meta-analyses on the management of OP poisoning up to the present.^14^,^15^ Our review will allow physicians a thorough and updated understanding of current management practices, evidence gaps, and future directions in treating OP poisoning.

## METHODS

We performed an umbrella review of the published systematic reviews and meta-analyses following the Preferred Reporting Items for Systematic reviews and Meta-Analyses methods^16^ using the protocol published in PROSPERO (CRD42025635860).

### Objective

Our goal was to critically review and summarize the available evidence from published systematic reviews and meta-analyses for the effect of various treatments on the outcomes of OP self-poisoning.

### Eligibility Criteria

#### Inclusion Criteria

We included systematic reviews and meta-analyses focusing on the management of OP self-poisoning. Studies analysing interventions such as atropine, pralidoxime, and novel therapies were eligible, provided they addressed the acute management of OP poisoning. Systematic reviews and meta-analyses were included irrespective of the year of publication.

#### Exclusion Criteria

We excluded case reports, observational studies, animal studies, and reviews that focused solely on the toxicology or epidemiology of OPs without addressing treatment. Studies published in languages other than English were also excluded unless translations were available.

### Databases Searched

The databases searched included PubMed, Epistemonikos, and the Cochrane Library. The search was carried out on January 7, 2025.

### Search Strategy

The keyword ‘Organoph’* was used in the title or abstract to capture variations such as “Organophosphorus,” “OP,” and “OPs.” We applied systematic review filters to refine the searches.

#### PubMed Search String

We used the following search terms: ((organophosphate[Medical Subject Headings (MeSH) Terms]) OR (Organoph*)) AND (systematic review) (“organophosphates”[MeSH Terms] OR “organoph*”[All Fields]) AND (“systematic review”[Publication Type] OR “systematic reviews as topic”[MeSH Terms] OR “systematic review”[All Fields])

#### Epistemonikos Search String

(title:(Organoph*) OR abstract:(Organoph*)) Filter: Systematic review.

#### Cochrane Search String

Organoph* (We searched word variations.)

### Data Management

#### Screening Process

Two reviewers (VC and ST) independently reviewed the titles and abstracts of all 488 articles, followed by full-text review of 27 selected articles for relevance based on the inclusion and exclusion criteria. Discrepancies were resolved through discussion with a third reviewer (SG).

### Data Extraction

Data extraction of the 19 included studies was conducted independently by two reviewers (VC and TP), using a pre-designed form. Extracted data included the following:

- Authors, year of publication, and journal title
- Study design (systematic reviews, meta-analyses)
- Types of interventions reviewed (eg, atropine, pralidoxime, novel therapies)
- Outcomes such as mortality, intermediate syndrome, and intubation/ventilation
- Pooled estimates (eg, relative risk, odds ratio)
- Recommendations and conclusions.

### Quality Assessment

The quality of included systematic reviews and meta-analyses was evaluated by two reviewers (ST and DG) independently using AMSTAR 2.^14^ We used the GRADE approach to assess the quality of evidence for each intervention.^15^ The GRADE was also applied independently by VC and TP, and they resolved any discrepancy in AMSTAR 2 and GRADE rating through discussion with the third reviewer SG.

### Data Synthesis

We performed a narrative synthesis of findings, grouping evidence by intervention and key outcome measures, such as mortality, intermediate syndrome, and intubation/ventilation.

### Primary Objective

We sought to generate recommendations and to grade the available evidence from published systematic reviews and meta-analyses for the effect of various treatments on the outcomes of OP self-poisoning.

### Secondary Objective

Our secondary goal was to highlight gaps in the current evidence base and identify emerging or future therapeutic strategies in OP poisoning management.

### Ethical Considerations

As this was an umbrella review of previously published research, institutional board approval was not required.

## RESULTS

### Literature Search

**Figure 1:**
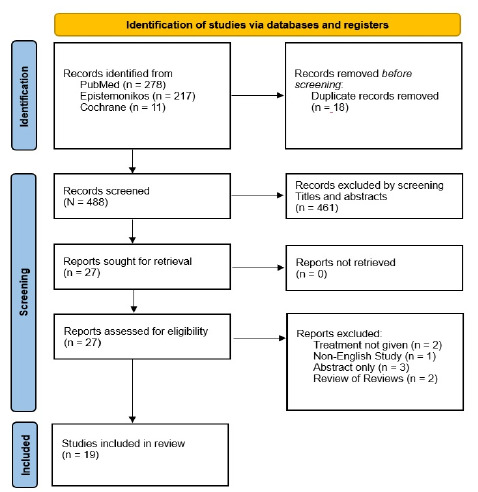
PRISMA* flow diagram for systematic reviews identified via databases on organophosphate self-poisoning. *PRISMA, Preferred Reporting Items for Systematic reviews and Meta-Analyses.

Our initial search found 206 results in PubMed, 217 in Epistemonikos, and 11 Cochrane reviews, which we imported into EndNote reference management software. Of these 428 papers, 18 were found to be duplicates, leaving a total of 416 for the screening and eligibility stages (Figure 1). Of the 416 papers screened, we excluded 389 that did not meet the inclusion criteria, leaving us with 27 papers for retrieval. We reviewed these 27 full texts for eligibility, which resulted in the exclusion of three that were only abstracts, two papers that did not review treatment of OP poisoning, one that was not available in English, and two that were reviews of previously published systematic reviews. This left a total of 19 papers for the final review. The process of screening and selecting studies is shown in the PRISMA flow diagram (Figure 1).

### Characteristics of Included Systematic Reviews

We included 19 systematic reviews and meta-analyses in this umbrella review, which describe 11 treatment options for OP self-poisoning (Table 1).

The oldest of the systematic reviews and meta-analyses was published in 2002 and the most recent in 2023. Many treatments were reported from China only; therefore, some systematic reviews and meta-analyses contained randomized controlled trials from one country alone. These treatments include penehyclidine, hemoperfusion + hemofiltration, lipid emulsion infusion, rhubarb, and xuebijing (which has not been reported outside China in any of the included trials.^7^–^10^, ^24^ No systematic reviews and meta-analyses studied the effectiveness of atropine, as it is the mainstay of treatment of OP poisoning and it would have been unethical to withhold this life-saving drug from the control group g. Most of the therapies looked at the additional benefit beyond that of atropine.

### Summary of Pooled Estimates

**Figure 2:**
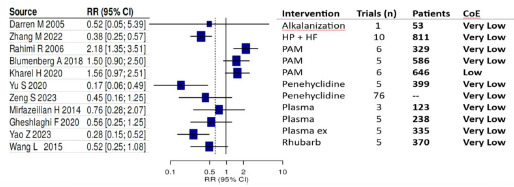
Summary of the pooled relative risk of mortality reported by the systematic reviews and metanalyses on various interventions in organophosphate self-poisoning. *RR*, relative risk; *CI*; confidence interval; *CoE*, Certainty of Evidence; *HP+HF*, Hemoperfusion + Hemofiltration; *PAM*, Pralidoxine; *Ex*, exchange.

**Figure 3:**
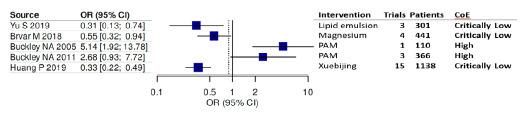
Summary of the pooled odds ratios of mortality reported by the systematic reviews and meta-analyses on various interventions in organophosphate self-poisoning. *CoE*, certainty of evidence; *OR;* odds ratio*; PAM*, pralidoxine.

Sixteen systematic reviews reported relative risk or odds ratio of mortality for 10 different interventions in the management of OP self-poisoning (Figures 2, 3). The figures also show the number of trials included in the systematic reviews and meta-analyses, total number of patients covered and certainty of evidence for each, ascertained using GRADE.

### Quality Assessment Using A Measurement Tool to Assess Systematic Reviews

Two reviewers independently rated the included systemic reviews using AMSTAR 2, and any differences were resolved through discussion with a third independent reviewer.^14^ See Table 2 for the AMSTAR 2 rating given to the included systematic reviews and meta-analyses.

### Review Questions and Recommendations for Treatment Options for OP Poisoning

#### Review Question 1

What is the effect of oximes on the outcomes of OP poisoning?

#### Recommendation Statement 1

Given the low certainty of evidence, it is not possible to definitively recommend or reject the use of oximes in the management of OP self-poisoning. Further well-designed, adequately powered trials are needed to clarify their role (Table 3a). (Evidence Overview: Supplement 1.1).

#### Review Question 2

What is the effect of plasma transfusion on the outcomes of OP poisoning?

#### Recommendation Statement 2

We conditionally recommend against the routine use of plasma transfusion in the management of OP self-poisoning (weak recommendation, GRADE: low certainty) (Table 3b) (Evidence Overview: Supplement 1.2).

#### Review Question 3

What is the effect of plasma exchange combined with hemoperfusion on the outcomes of OP poisoning?

#### Recommendation Statement 3

We conditionally recommend against the routine use of plasma exchange combined with hemoperfusion in the management of OP self-poisoning (Weak recommendation, GRADE: very low certainty) (Table 3c) Evidence Overview: Supplement 1.3).

#### Review Question 4

What is the effect of hemoperfusion with hemofiltration on mortality, intermediate syndrome, and intubation/ventilation in patients with OP poisoning?

#### Recommendation Statement 4

**We conditional**ly recommend against the routine use of hemoperfusion with hemofiltration in the management of OP self-poisoning (weak recommendation, GRADE: ery low certainty) (Table 3d) (Evidence Overview: Supplement 1.4).

#### Review Question 5

What is the effect of lipid emulsion on mortality in patients with OP poisoning?

#### Recommendation Statement 5

We conditionally recommend against the routine use of lipid emulsion in the management of OP self-poisoning (weak recommendation, GRADE: very low certainty) (Table 3e) (Evidence Overview: Supplement 1.5).

#### Review Question 6

What is the effect of magnesium sulfate on the outcomes of OP poisoning?

#### Recommendation Statement 6

We conditionally recommend against the routine use of magnesium sulfate in the management of OP self-poisoning (Weak recommendation, GRADE: very low certainty) (Table 3f) Evidence Overview: Supplement 1.6).

#### Review Question 7

What is the effect of gastric lavage on the outcomes of OP poisoning?

#### Recommendation Statement 7

We conditionally recommend against gastric lavage in the management of OP self-poisoning. (Weak recommendation, GRADE: very low certainty) (Evidence Overview: Supplement 1.7)

#### Review Question 8

What is the effect of alkalinization on the outcomes of OP poisoning?

#### Recommendation Statement 8

We conditionally recommend against the routine use of alkalinization in the management of OP self-poisoning (Weak recommendation, GRADE: very low certainty) (Table 3g) Evidence Overview: Supplement 1.8).

#### Review Question 9

What is the effect of penehyclidine on the outcomes of OP poisoning?

#### Recommendation Statement 9

We conditionally recommend against the use of penehyclidine in the management of OP self-poisoning based on very low certainty of evidence. Further high-quality RCTs are needed to confirm its effectiveness and safety (Table 3h*)* (Evidence Overview: Supplement 1.9).

#### Review Question 10

What is the effect of rhubarb on the outcomes of OP poisoning?

#### Recommendation Statement 10

We conditionally recommend against the use of rhubarb in the management of OP self-poisoning based on very low certainty evidence. High-quality, adequately powered RCTs are needed to confirm its effectiveness and safety (Table 3i) (Evidence Overview: Supplement 1.10).

#### Review Question 11

What is the effect of xuebijing on the outcomes of OP poisoning?

#### Recommendation Statement 11

We conditionally recommend against the use of xuebijing in the management of OP self-poisoning based on very low certainty evidence. Higher quality, adequately powered RCTs are required to establish its effectiveness and safety. (Table 3j) Evidence Overview: Supplement 1.11

## DISCUSSION

Organophosphate self-poisoning is a critical global health concern, particularly in agricultural regions where OP pesticides are prevalent. Despite the availability of treatments such as atropine and oximes, the effectiveness of many therapeutic interventions remains uncertain. This comprehensive review of systematic reviews and meta-analyses highlights the current state of evidence for treatments including oximes, plasma exchange, hemoperfusion, lipid emulsions, magnesium sulfate, gastric lavage, alkalinization, penehyclidine, rhubarb, and xuebijing. However, significant gaps and limitations persist across studies, necessitating further research.

Oximes, such as pralidoxime, are recommended by the World Health Organization and are widely used in OP poisoning, but their efficacy remains debatable. The most recent systematic reviews and meta-analysis by Kharel et al (2020) pooled six RCTs and found no clear evidence of benefit or harm, with low-quality evidence suggesting a relative risk of mortality of 1.53.^4^ Methodological flaws, including inadequate sample sizes, inconsistent dosing, and variability in the OP compounds studied limit the reliability of conclusions made by the systematic reviews and meta-analyses on oximes.

Gastric lavage, traditionally used to remove ingested toxins, also lacks robust evidence.^26^ Reviews have failed to demonstrate significant benefits due to the absence of control groups and variability in lavage protocols, leaving its role in OP poisoning uncertain. The recommendations are to not perform gastric lavage beyond one hour of OP self-poisoning as the benefits are uncertain and may even result in harm.

Plasma exchange and hemoperfusion, aimed at enhancing toxin elimination, show potential benefits in isolated studies but lack high-quality evidence. The systematic reviews and meta-analysis by Yao et al (2023) suggested that plasma exchange with hemoperfusion may reduce mortality, but we found that the conclusions were drawn based on studies with very low certainty of evidence, geographic bias, and poor study designs.^12^ We need properly designed studies on plasma exchange and hemoperfusion in OP poisoning and, therefore, we recommend against their routine use.

Lipid emulsions have been explored for their ability to sequester lipophilic toxins, with some studies indicating reduced mortality in OP poisoning.^10^ However, the evidence is weak due to small sample sizes, variability in protocols, and critically low-quality ratings in the reviews, and, therefore, we recommend not to use them routinely.

Magnesium sulfate, another potential treatment, has shown some promise in reducing mortality and ventilation requirements, but inconsistent dosages and methodological flaws limit its utility.^6^ We recommend not to use magnesium sulfate for OP self-poisoning. Alkalinization, primarily using sodium bicarbonate, has been studied for its potential to enhance toxin clearance.^25^ However, evidence remains weak, with reviews failing to demonstrate significant clinical benefits. We recommend against the use of alkalanization for OP self-poisoning.

Penehyclidine, an anticholinergic drug, has shown promise in improving outcomes when used along with atropine, although the studies pooled to generate this evidence have critical methodological flaws and geographic bias.^9^ We recommend against the use of penehyclidine in OP self-poisoning. Finally, rhubarb and xuebijing, both herbal treatments commonly used in China, have also demonstrated potential benefits as adjunctive therapy, but they too face similar challenges of limited generalizability and poor quality of the evidence.^7^,^8^ We recommend against the use of rhubarb and xuebijing in patients with OP self-poisoning.

## SUMMARY of LIMITATIONS of REVIEWED STUDIES

We found limitations of the current evidence on plasma transfusion, oximes, and other treatments for OP poisoning. These limitations include methodological flaws such as small sample sizes, lack of randomization, and inconsistent reporting of outcomes. Many studies were conducted only in China, which raises concerns about geographical and publication bias. In addition, several trials did not provide details on the type or dose of OPs involved, limiting the applicability of findings. The certainty of evidence was generally rated as very low, with several reviews receiving a critically low **AMSTAR-2** rating, indicating that the available data cannot be relied upon for definitive conclusions.

## RECOMMENDATIONS for FUTURE TRIALS

Future studies should focus on large, well-designed RCTs with sufficient sample sizes to test the effectiveness of various treatments in OP poisoning. Trials should aim to detect the impact of interventions such as magnesium sulfate, penehyclidine, rhubarb, xuebijing, lipid emulsion, plasma transfusion, hemoperfusion, gastric lavage, alkalinization, and oximes on key clinical outcomes, including mortality, intermediate syndrome, intubation/ventilation rates, and the need for specific therapies. Adequately powered RCTs will ensure robust and reliable findings that contribute to better treatment protocols. By addressing these recommendations, future research can contribute to more conclusive evidence on the most effective treatments for OP poisoning, improve clinical practice, and ultimately enhance patient outcomes.

## CONCLUSION

After careful scrutiny of the current evidence pooled by various systematic reviews and meta-analyses we conclude that atropine remains the mainstay of treatment for OP self-poisoning. It may be supplemented with oximes as recommended by the WHO despite no clear evidence for benefit or harm from the systematic reviews and meta-analyses. Gastric lavage has doubtful efficacy and may even be harmful if done beyond one hour of poisoning. Additionally, we recommend against the routine use of penehyclidine, rhubarb, xuebijing, hemofiltration, plasma exchange with hemoperfusion, lipid emulsions, magnesium sulfhate and alkalinization in the management of OP self-poisoning. Overall, the evidence for the treatments for OP self-poisoning, other than atropine, is plagued by methodological weaknesses, small sample sizes, and biases. Many studies lack rigorous randomization, blinding, and standardized protocols, reducing the reliability of their findings. Additionally, the geographic concentration of research, particularly in China, raises concerns about the generalizability of results to other populations.

## Supplementary Information



## Figures and Tables

**Table 1:** Treatment options in a systematic review of organophosphate self-poisoning.

Sr. No	Treatment option	Reviews (n)	Patient (n)	Author, year
1	Oximes (Pralidoxime)	7	646	Eddleston, 2002^17^; Buckley, 2005^18^; Peter, 2006^19^; Rahimi, 2006^20^; Buckley, 2011^3^; Blumenberg, 2018^21^; Kharel 2020^4^
2	Plasma transfusion	2	299	Mirfazaelian,2014^22^; Gheshlaghi, 2020^23^
3	Penehyclidine	3	20,797	Yu, 2020^9^; Zeng, 2023^11^
4	Plasma exchange + Hemoperfusion	1	1,034	Yao, 2023^12^
5	Hemoperfusion + hemofiltration	1	811	Zhang, 2022^24^
6	Alkalinization	1	133	Darren, 2005^25^
7	Lipid emulsion infusion	1	630	Yu, 2019^10^
8	Gastric lavage	1	4,288	Li, 2009^26^
9	Magnesium sulfate	1	441	Brvar, 2018^6^
10	Rhubarb	1	886	Wang, 2015^7^
11	Xuebijing	1	1,880	Huang 2019^8^

**Table 2:** Results of the quality assessment of included systematic reviews with the AMSTAR 2^*^ tool.

REFERENCE	1. PICO	2. A priori study Protocol	3. Choice study design	4. Search strategy	5. Duplicate study selection	6. Duplicate data extraction	7. List of excluded studies	8. Description included studies	9. Technique RoB	10. Funding sources	11. statistical methods	12. Impact of RoB on results	13. RoB discussion	14. Heterogeneity discussion	15. Publication bias	16. Conflict of interest	Overall score
BLUMENBERG 2018	Y	Y	N	PY	Y	Y	N	PY	Y	N	Y	N	Y	Y	Y	Y	Low
BRVAR 2018	Y	N	N	PY	Y	Y	N	PY	Y	N	Y	N	N	Y	N	Y	Critically Low
BUCKLEY 2005	Y	Y	Y	Y	Y	Y	Y	Y	Y	N	Y	Y	Y	Y	Y	Y	High
BUCKLEY 2011	Y	Y	Y	Y	Y	Y	Y	Y	Y	Y	Y	Y	Y	Y	Y	Y	High
DARREN 2005	Y	Y	Y	PY	N	Y	Y	Y	Y	N	NM	NM	Y	Y	NM	Y	Moderate
EDDLESTON 2002	N	N	N	PY	N	N	N	PY	N	N	NM	NM	N	N	NM	Y	Critically Low
GHESHLAGHI 2020	Y	N	N	PY	Y	Y	N	PY	N	Y	Y	N	N	Y	Y	Y	Critically Low
HUANG 2019	Y	N	N	PY	Y	Y	N	PY	Y	N	Y	N	Y	Y	Y	Y	Critically Low
KHAREL 2020	Y	Y	N	Y	Y	Y	N	PY	Y	N	Y	N	Y	Y	Y	Y	Low
LI 2009	N	N	N	PY	Y	Y	N	PY	PY	N	NM	NM	Y	Y	NM	N	Critically Low
MIRFAZAELIAN 2014	Y	N	N	PY	Y	N	N	PY	N	N	Y	N	N	Y	N	Y	Critically Low
PETER 2006	Y	N	N	PY	N	N	N	PY	N	N	Y	Y	N	N	N	N	Critically Low
RAHIMI 2006	Y	N	N	N	Y	Y	N	N	N	N	N	N	N	N	Y	N	Critically Low
WANG 2015	Y	N	N	PY	N	Y	N	PY	PY	N	Y	N	N	Y	Y	Y	Critically Low
YAO 2023	Y	N	N	PY	N	Y	N	PY	Y	N	Y	N	N	Y	Y	Y	Critically Low
YU 2018	Y	N	N	PY	Y	Y	N	PY	Y	N	Y	N	N	Y	Y	Y	Critically Low
YU 2020	Y	N	N	PY	Y	Y	N	PY	Y	N	Y	N	N	Y	Y	Y	Critically Low
ZENG 2023	Y	Y	N	PY	Y	Y	N	PY	Y	N	Y	N	N	Y	Y	Y	Critically Low
ZHANG 2022	Y	N	N	PY	Y	Y	N	PY	Y	N	Y	N	N	Y	Y	Y	Critically Low

High: No or one non-critical weakness: the systematic review provides an accurate and comprehensive summary of the results of the available studies that address the question of interest. Moderate: More than one non-critical weakness*: the systematic review has more than one weakness but no critical flaws. It may provide an accurate summary of the results of the available studies that were included in the review. Low: One critical flaw with or without non-critical weaknesses: the review has a critical flaw and may not provide an accurate and comprehensive summary of the available studies that address the question of interest Critically low: More than one critical flaw with or without non-critical weaknesses: the review has more than one critical flaw and should not be relied on to provide an accurate and comprehensive summary of the available studies																	

AMSTAR, A Measurement Tool to Assess Systematic Reviews; NM, no meta-analysis done; PICO, population intervention comparator outcome; PY, partial yes; ROB, risk of bias.

**Table 3a:** Certainty of evidence for the use of Pralidoxime for organophosphate poisoning, Kharel 2020.

Outcomes	Anticipated absolute effects^*^ (95% CI)		Relative effect (95% CI)	№ of participants (studies)	Certainty of the evidence (GRADE)	Comments
	Risk with Placebo	Risk with Pralidoxime				
Mortality	141 per 1,000	**215 per 1,000** (136 to 339)	**RR 1.53** (0.97 to 2.41)	646 (6 RCTs)	⊕ ⊚⊚ Low^a^,^b^,^c^,^d^,^e^	
Intermediate syndrome	267 per 1,000	**435 per 1,000** (269 to 699)	**RR 1.63** (1.01 to 2.62)	210 (2 RCTs)	⊕⊚⊚⊚ Very low^f^,^g^,^h^,^i^	
Intubation/ ventilation	269 per 1,000	**347 per 1,000** (261 to 460)	**RR 1.29** (0.97 to 1.71)	646 (6 RCTs)	⊕ ⊚⊚ Low^a^,^b^,^c^,^d^	

a Three new studies were added to this meta-analysis after the 2011 Cochrane review by Bukley et al. The earlier 3 studies had very low grade, which persists for the more recent 3 studies included in this review.

b The 3 new studies added to this review had risk of other bias due to lack of sample size, power calculation, and stopping rule.

c The 2 new studies, both by Banerjee et al, were open-label studies.

d The point estimates of 3 of 5 studies lie outside the 95% CI of the largest and most well-conducted study in this review

e Cherian 1997 blinding was unclear.

f Only 2 small studies contributing; while Cherian 1997 has a statistically significant result, the confidence interval is wide.

g Only 2 small studies provide this outcome.

h Point estimate of Syed et al lies outside 95% CI of Cherian et al.

i Cherian 1997 was a small study with substantial risk of bias. Low dose of pralidoxine given may lack efficacy.

**Table 3b:** Certainty of evidence for the use of fresh frozen plasma for organophosphate poisoning, Gheshlaghi 2020.

Outcomes	Anticipated absolute effects^*^ (95% CI)		Relative effect (95% CI)	№ of participants (studies)	Certainty of the evidence (GRADE)	Comments
	Risk with Placebo	Risk with Fresh Frozen Plasma				
Death	132 per 1,000	**74 per 1,000** (33 to 167)	**RR 0.56** (0.25 to 1.26)	238 (5 RCTs)	⊕ ⊚⊚ Low^a^,^b^,^c^,^d^	
Intubation/Ventilation	379 per 1,000	**508 per 1,000** (247 to 1,000)	**RR 1.34** (0.65 to 2.74)	48 (2 RCTs)	⊕⊚⊚⊚ Very low^a^,^b^,^c^,^e^,^f^	

a All were unblinded studies.

b One of the studies was partially randomized.

c All studies had small sample sizes.

d Studies had wide confidence intervals, and their point estimates lie quite away from the pooled estimate on both sides.

e Point estimate of one study lies outside confidence interval of the other study.

f Only 2 studies included in the pooled estimate.

*GRADE*, Grading of Recommendations Assessment, Development and Evaluation; *RCT*, randomized controlled trial; *RR*, relative risk.

**Table 3c:** Certainty of evidence for the use of plasma exchange plus hemoperfusion vs. hemoperfusion for organophosphate poisoning, Yao 2023.

Outcomes	Anticipated absolute effects^*^ (95% CI)		Relative effect (95% CI)	№ of participants (studies)	Certainty of the evidence (GRADE)	Comments
	Risk with Hemoper fusion	Risk with Plasma Exchange plus Hemoperfusion				
Mortality	231 per 1,000	**65 per 1,000** (35 to 120)	**RR 0.28** (0.15 to 0.52)	335 (5 RCTs)	⊕⊚⊚⊚ Very low^a^,^b^,^c^,^d^,^e^,^f^	

a None of the studies reported allocation concealment, blinding of participants, and blinding of outcomes; 3 of 5 studies did not report using random sequence generation. All were classified as unclear for selective reporting and other bias by the author of the systematic review.

b The control group treatments are inconsistent among the studies. Atropine use was mentioned in only 2 of 5 studies for the control and experimental groups.

c One study did not use gastric lavage and diuresis, while 4 others did in both groups.

d 2 of 5 studies used catharsis in both groups.

e 1 of 5 studies used phosphoridine in both the groups.

f The interventions in the control and experimental groups do not match, therefore could not be combined to get pooled estimates.

*GRADE*, Grading of Recommendations Assessment, Development and Evaluation; *RCT*, randomized controlled trial; *RR*, relative risk.

**Table 3d:** Certainty of evidence for the use of hemofiltration with hemoperfusion for organophosphate poisoning, Zhang M 2022.

Outcomes	Anticipated absolute effects^*^ (95% CI)		Relative effect (95% CI)	№ of participants (studies)	Certainty the evidence (GRADE)	Comments
	Risk with Placebo	Risk with Hemofiltration with Hemoperfusion				
Death	195 per 1,000	**74 per 1,000** (49 to 111)	**RR 0.38** (0.25 to 0.57)	811 (10 RCTs)	⊕⊚⊚⊚ Very low^a^,^b^,^c^,^d^	

a None of the 10 studies reported allocation concealment.

b Only 1 of the 10 studies reported blinding to allocation and outcomes.

c 5 of 10 studies did not use random sequence generation.

d There were gross differences in the control groups: in 6 studies the control groups received hemoperfusion + conventional treatment; 1 received hemofiltration + conventional treatment, and 3 received only conventional treatments.

*GRADE*, Grading of Recommendations Assessment, Development and Evaluation; *RR*, relative risk; *RCT*, randomized controlled trial.

**Table 3e:** Certainty of evidence for the use of lipid emulsion for organophosphate poisoning, Yu 2019.

Outcomes	Anticipated absolute effects^*^ (95% CI)		Relative effect (95% CI)	№ of participants (studies)	Certainty of the evidence (GRADE)	Comments
	Risk with Placebo	Risk with Lipid Emulsion				
Mortality	136 per 1,000	**47 per 1,000** (20 to 105)	**OR 0.31** (0.13 to 0.74)	301 (4 RCTs)	⊕⊚⊚⊚ Very low^a^,^b^,^c^,^d^,^e^	

a None of the studies had allocation concealment, blinding of participants, personnel or outcome assessors.

b One of the studies did not mention random sequence generation.

c One of the studies did not report mortality in either of the groups,

d Four studies used lipid emulsion for different durations: 3, 5, 6, and 7 days, respectively.

*GRADE*, Grading of Recommendations Assessment, Development and Evaluation; *OR*, odds ratio; *RCT*, randomized controlled trial.

**Table 3f:** Certainty of evidence for the use of magnesium sulfate for organophosphate poisoning, Byrar 2018.

Outcomes	Anticipated absolute effects^*^ (95% CI)		Relative effect (95% CI)	№ of participants (studies)	Certainty of the evidence (GRADE)	Comments
	Risk with Placebo	Risk with Magnesium Sulphate				
Mortality	188 per 1,000	**113 per 1,000** (69 to 179)	**OR 0.55** (0.32 to 0.94)	441 (8 RCTs)	⊕⊚⊚⊚ Very low^a^,^b^,^c^	
Intubation/Ventilation	475 per 1,000	**320 per 1,000** (235 to 417)	**OR 0.52** (0.34 to 0.79)	377 (6 RCTs)	⊕⊚⊚⊚ Very low^a^,^b^,^c^	

a Authors combined case series before-and-after studies with RCTs in the meta-analysis.

b Most of the included studies had small numbers, and no power or sample-size calculations.

c Dose of magnesium sulfate was variable among the studies.

*GRADE*, Grading of Recommendations Assessment, Development and Evaluation; *OR*, odds ratio; *RCT*, randomized controlled trial.

**Table 3g:** Certainty of evidence for the use of alkalinization for organophosphate poisoning, Darren 2005.

Outcomes	Anticipated absolute effects^*^ (95% CI)		Relative effect (95% CI)	№ of participants (studies)
	Risk with placebo	Risk with Alkalinization		
Mortality	74 per 1,000	**39 per 1,000** (4 to 399)	**RR 0.52** (0.05 to 5.39)	53 (1 RCT)
Intubation/Ventilation	37 per 1,000	**199 per 1,000**	**RR 0.50**	53
		(1 to 217)	(0.04 to 5.87)	(1 RCT)

a Author categorized alternate admissions to two groups, not properly randomized, no concealment, no blinding.

b Confidence interval was very wide.

c Single study included in the analysis; sample size was small.

*RR*, relative risk; *RCT*, randomized controlled trial.

**Table 3h:** Certainty of evidence for the use of penehyclidine for organophosphate poisoning, Yu 2020.

Outcomes	Anticipated absolute effects^*^ (95% CI)		Relative effect (95% CI)	№ of participants (studies)	Certainty of the evidence (GRADE)	Comments
	Risk with Placebo	Risk with Penehyclidine				
Mortality	111 per 1,000	**19 per 1,000** (7 to 54)	**RR 0.17** (0.06 to 0.49)	399 (5 RCTs)	⊕⊚⊚⊚ Very low^a^,^b^,^c^,^d^	

a None of the 5 studies reported allocation concealment, blinding of participants, workers or outcome assessors.

b All studies were from one country only.

c Dose of the experimental drug varied from 1 mg three time daily to 4–6 mg stat. Dosage was not same in any 2 studies.

*GRADE*, Grading of Recommendations Assessment, Development and Evaluation; *RR*, relative risk; *RCT*, randomized controlled trial.

**Table 3i:** Certainty of evidence for the use of crude rhubarb for organophosphate poisoning, Wang 2015.

Outcomes	Anticipated absolute effects^*^ (95% CI)		Relative effect (95% CI)	№ of participants (studies)	Certainty of the evidence (GRADE)	Comments
	Risk with Placebo	Risk with Crude rhubarb				
Mortality	95 per 1,000	**49 per 1,000** (24 to 103)	**RR 0.52** (0.25 to 1.08)	370 (5 RCTs)	⊕⊚⊚⊚ Very low^a^,^b^,^c^,^d^,^e^,^f^,^g^	
Intermediate syndrome	142 per 1,000	**31 per 1,000** (14 to 68)	**RR 0.22** (0.10 to 0.48)	434 (6 RCTs)	⊕⊚⊚⊚ Very low^a^,^b^,^c^,^d^,^e^,^f^	

a Although most of the studies were categorized as RCTs, only one provided method of randomization.

b None of the studies reported allocation concealment.

c None of the studies reported blinding of participants, workers, or outcome assessors.

d All studies were from one country only.

e The treatments given in the control and intervention groups were not similar. Use of catharsis and diuretics was variable in both groups across studies.

*GRADE*, Grading of Recommendations Assessment, Development and Evaluation; *RR*, relative risk; *RCT*, randomized controlled trial.

**Table 3j:** Certainty of evidence for the use of xuebijing for organophosphate poisoning, Huang 2019.

Outcomes	Anticipated absolute effects* (95% CI)			№ of participants (studies)	Certainty of the evidence (GRADE)	Comments
	Risk with placebo	Risk with xuebijing	Relative effect (95% CI)			
Mortality	174 per 1,000	**65 per 1,000** (44 to 94)	**OR 0.33** (0.22 to 0.49)	1138 (15 RCTs)	⊕⊚⊚⊚ Very low^a^,^b^,^c^	

a Most of the studies lacked allocation concealment, and blinding.

b Two studies used blood perfusion as an additional intervention in the trial group, which should not have been included in the pooled estimate.

c All studies are from a single country.

* The risk in the intervention group (and its 95% CI) is based on the assumed risk in the comparison group and the relative effect of the intervention (and its 95% CI).

GRADE Working Group grades of evidence:

High certainty: We are very confident that the true effect lies close to that of the estimate of the effect.

Moderate certainty: We are moderately confident in the effect estimate; the true effect is likely to be close to the estimate of the effect, but there is a possibility that it is substantially different.

Low certainty: Our confidence in the effect estimate is limited; the true effect may be substantially different from the estimate of the effect.

Very low certainty: We have very little confidence in the effect estimate; the true effect is likely to be substantially different from the estimate of effect.

*GRADE*, Grading of Recommendations Assessment, Development and Evaluation; *RR*, relative risk, *RCT*, randomized controlled trial.
