# Supplementary material for: Therapeutic Interventions in Organophosphate Poisoning: An Umbrella Review of Systematic Reviews
Source: West J Emerg Med. 2026 May 19;27(3):819–30. doi: 10.5811/westjem.50823 (PMC13246183; doi:10.5811/westjem.50823)
Supplement: Supplementary file 1 [file wjem-27-819-s001.docx]

Supplementary file 1

**Supplement 1.1:**

This question has been addressed by **7 SRMAs** published between **2002 and 2020**, exploring the use of **oximes** in **Organophosphate poisoning**. The reviews were conducted by various authors, with notable contributions from **Eddleston et al. (2002)**, **Buckley et al. (2005, 2011)**, **Peter et al. (2006)**, **Rahimi et al. (2006)**, **Blumenberg et al. (2018)**, and **Kharel et al. (2020)**.

1. **Eddleston et al. (2002)**: No meta-analysis was conducted due to a lack of high-quality RCTs. (11)
2. **Buckley N. (2005)**: The **Cochrane review** included only one RCT by **Cherian et al.** (25), which suggested that oximes might be harmful in OP poisoning. This review, despite being of GRADE **High quality**, called for larger, more specific trials to accurately assess the benefit of oximes. (12)
3. **Peter et al. (2006) and Rahimi et al. (2006)**: These SRMAs combined **RCTs and non-RCTs**, leading to **methodological flaws** and **critically low quality** of evidence. (13, 14)
4. **Buckley et al. (2011)**: This review updated the evidence, including **3 RCTs**. (25-27) It still found insufficient evidence to conclude whether oximes were beneficial or harmful, calling for **larger, higher-quality studies**. (3)
5. **Blumenberg et al. (2018)**: This review added **2 trials** to the previous data, but still could not determine if oximes were beneficial or harmful. (15)
6. **Kharel et al. (2020)**: The most recent SRMA included **6 RCTs** with a total of **646 patients** (326 in the pralidoxime group and 320 in the placebo group). (4) It found that the **relative risk of mortality** was **1.53 (95% CI: 0.97-2.41)**, suggesting a potential **increase in mortality** with oximes, but the evidence was **low certainty** due to methodological flaws and inconsistent reporting. **GRADE for certainty of evidence**: **Low** (Table 3a) Due to the methodological flaws of the RCTs, **lack of sufficient data**, and the **variability** in the type of **Organophosphate** compounds and doses used, the certainty of the evidence was low. This SRMA itself received an **AMSTAR-2 rating of ‘low’**, indicating that the review has a critical flaw and may not provide an accurate and comprehensive summary of the available studies that address the question of interest (Table 2).

**Supplement 1.2:**

Two **SRMAs** addressed this question:

1. **Mirfazaelian et al. (2014)** (16)

This review included **3 RCTs** by **Guven et al**, **Pichamuthu et al**, and **Pazooki et al**. (28-30)

1. **Gheshlaghi et al. (2020)** (updated SRMA) (5)

**This review** updated the previous review by adding **2 more RCTs** by **Sameh et al** and **Dayananda et al**. (31, 32)

- **Plasma transfusion** is thought to work by utilizing the **butylcholinesterase enzyme** present in plasma, which can bind free **Organophosphate** in the blood and facilitate its removal.
- The **SRMAs** did not find a **significant effect** of plasma transfusion in addition to conventional therapy on **mortality** or **Intermediate Syndrome** (IMS) in **Organophosphate** poisoning patients.
- Both SRMAs received an **AMSTAR-2 rating of ‘Critically low’ (Table 2)**, signalling concerns about the **quality** and **completeness** of the included studies.
- The **GRADE** assessment also concluded a **low certainty of evidence** due to the limitations mentioned. (Table 3b)

**Supplement 1.3:**

One **SRMA** addressed this question:

1. Yao et al. (2023) (18)

- This systematic review and meta-analysis (SRMA) included five randomized controlled trials (RCTs). (18) Their findings demonstrated a significant benefit of plasma exchange combined with hemoperfusion on clinical outcomes in OP poisoning.
- However, the certainty of evidence (GRADE) was rated as **‘very low’** (Table 3c), due to several limitations, and the SRMA itself received an **AMSTAR-2 rating of ‘Critically low’**, indicating a lack of reliability in summarizing available studies (Table 2).

**Supplement 1.4:**

One **SRMA** addressed this question:

1. Zhang et al. (2022) (13)

- The review included ten randomized controlled trials (RCTs), all of which were from a single country, China. The RCTs included in the review had poor methodological quality, and the control groups in the studies were quite dissimilar in the treatments they received. Notably, seven out of the ten RCTs also gave hemoperfusion to the control groups, which creates a potential bias in estimating the true effect of hemoperfusion with hemofiltration.
- Despite reporting a significant benefit of hemoperfusion with hemofiltration in reducing mortality in **Organophosphate** poisoning, the certainty of this evidence was rated as **‘very low’** according to the GRADE assessment (Table 3d). Furthermore, the SRMA received an **AMSTAR-2 rating of ‘Critically low’**, which means that it should not be relied upon for accurate or comprehensive conclusions (Table 2).

**Supplement 1.5:**

One **SRMA** addressed this question:

1. Yu et al. (2019) (21)

- The review included four randomized controlled trials (RCTs) that used 20%-30% lipid emulsion at a dose of 250 ml once daily for a duration of 3-7 days, in combination with conventional treatment. The results showed a **significant reduction in mortality** with an odds ratio (OR) of **0.31** (95% CI: 0.13 to 0.74), indicating a potential benefit of lipid emulsion in reducing mortality in **Organophosphate** poisoning.
- However, despite the significant findings, the **GRADE rating for the certainty of evidence** was **‘very low’** (Table 3e), reflecting the high degree of uncertainty surrounding these results. Additionally, the SRMA received a **‘Critically low’** AMSTAR-2 rating, which indicates that the summary should not be relied upon for an accurate or comprehensive understanding of the available studies. (Table 2)

**Supplement 1.6:**

One **SRMA** addressed this question:

1. **Brvar et al. (2018)** (7)

- Magnesium sulfate is an inexpensive and widely available drug, and the review found that it significantly reduced both mortality and the need for intubation/ventilation in **Organophosphate** poisoning patients. However, the **certainty of evidence (GRADE)** was rated as **‘very low’**(Table 3f), due to several factors, including the inclusion of case series alongside randomized controlled trials (RCTs), which could have affected the validity of the conclusions. Additionally, the **AMSTAR-2 rating was ‘Critically low’**(Table 2), meaning that the SRMA should not be relied upon to provide an accurate and comprehensive summary of the available studies.
- The studies included in the SRMA used varying dosages of magnesium sulfate (some used a 4 g bolus, others used 4 g every 6 hours for 2 days), adding another layer of heterogeneity to the findings.

**Supplement 1.7:**

One SRMA addressed this question:

1. Li et al. (2009) (22)

- They found that no randomized controlled trials (RCTs) had a control arm without gastric lavage, and therefore no meta-analysis was performed.
- All included studies were from China, and they reported benefits of gastric lavage, including techniques such as multiple lavages, the use of norepinephrine or pralidoxime in lavage fluid, concurrent treatment with naloxone or scopolamine, and even the insertion of the gastric tube via a laparotomy incision or performing lavage later than 12 hours post-ingestion.
- The evidence was largely heterogeneous and lacked high-quality control groups.
- The **AMSTAR-2 rating was ‘Critically low’**(Table 2), meaning that the SRMA should not be relied upon to provide an accurate and comprehensive summary of the available studies.

**Supplement 1.8:**

One SRMA addressed this question:

1. Darren et al. (2005) (20)

- The authors identified only **one RCT** that reported on mortality and intubation/ventilation as outcomes of alkalinisation in addition to conventional treatment. The included study did not find any significant effect of alkalinisation on the outcomes of **Organophosphate** poisoning.
- Despite the **AMSTAR-2 rating of ‘Moderate’** (Table 2), for the review, the **GRADE** for the certainty of evidence was rated **‘very low’** (Table 3g), mainly due to the inclusion of only a single study in the analysis, limiting the ability to draw robust conclusions.

**Supplement 1.9:**

Two SRMAs addressed this question.

1. Yu et al. (2020) (5)

This SRMA included 5 RCTs, reporting a beneficial pooled effect of Penehyclidine on mortality in **Organophosphate** poisoning.

1. Zeng et al. (2023) (16)

This SRMA included 76 RCTs, but the studies included by Yu et al. were not part of this analysis, and there was no clear explanation for this large discrepancy in study inclusion, despite similar search strategies being used.

- Both SRMAs reported that Penehyclidine, when added to atropine, results in a significant improvement in **Organophosphate** poisoning outcomes.
- All studies in both SRMAs were from China.
- **AMSTAR-2 Rating**: Both reviews were rated ‘Critically low’, indicating significant concerns about the quality and comprehensiveness of the reviews. (Table 2),
- **GRADE**: The certainty of evidence for the SRMA by Yu et al. was rated **‘very low’** (Table 3h). No GRADE assessment was performed for Zeng et al. because outcome data was not provided by the authors.

**Supplement 1.10:**

**One SRMA addressed this question:**

1. **Wang et al. (2015)** (23)

The SRMA by Wang et al. included **12 RCTs** and found that rhubarb, when added to atropine, had **beneficial effects** on the outcomes of **Organophosphate** poisoning.

- Rhubarb is believed to have antioxidant activity, antiplatelet aggregation effects, and anti-inflammatory properties, which may contribute to improving the outcomes of **Organophosphate** poisoning.
- **Geographical Limitation:** All the included studies were from **China**, which raises concerns about the generalizability of the findings.
- **GRADE**: The certainty of evidence was rated **‘very low’** (Table 3i) due to various limitations in the included studies.
- **AMSTAR-2 Rating**: The review was rated **‘Critically low’** (Table 2), indicating significant concerns about the quality of the review and the studies included.

**Supplement 1.11:**

One SRMA addressed this question:

1. Huang et al. (2019) (24)

The SRMA by Huang et al. included **15 RCTs** and found a **significant clinical effect** of Xuebijing injection at a dose of **50 ml twice a day in addition to atropine** in improving outcomes of **Organophosphate** poisoning.

- **Xuebijing** is a **Chinese herbal patented medicine** with **anti-inflammatory, anti-oxidation, immune regulatory, and antiplatelet effects**.
- **Geographical Limitation:** All the studies included in the SRMA were from **China**, which limits the external validity of the findings.
- **GRADE**: The certainty of evidence was rated **‘very low’** (Table 3j) due to methodological weaknesses.
- **AMSTAR-2 Rating**: The SRMA was rated **‘Critically low’** (Table 2), signalling concerns about the quality of the included studies and the overall reliability of the findings.
